# Transgenic Cotton Plants Expressing Cry1Ia12 Toxin Confer Resistance to Fall Armyworm (*Spodoptera frugiperda*) and Cotton Boll Weevil (*Anthonomus grandis*)

**DOI:** 10.3389/fpls.2016.00165

**Published:** 2016-02-19

**Authors:** Raquel S. de Oliveira, Osmundo B. Oliveira-Neto, Hudson F. N. Moura, Leonardo L. P. de Macedo, Fabrício B. M. Arraes, Wagner A. Lucena, Isabela T. Lourenço-Tessutti, Aulus A. de Deus Barbosa, Maria C. M. da Silva, Maria F. Grossi-de-Sa

**Affiliations:** ^1^Catholic University of BrasiliaBrasilia, Brazil; ^2^Pest-Plant Molecular Interaction Laboratory, Embrapa Genetic Resources and Biotechnology, Brazilian Research Agricultural CorporationBrasilia, Brazil; ^3^UNIEURO – University CenterBrasília, Brazil; ^4^Biology Institute, Brasilia UniversityBrasilia, Brazil; ^5^Federal University of Rio Grande do SulPorto Alegre, Brazil; ^6^Embrapa CottonCampina Grande, Brazil

**Keywords:** *Gossypium hirsutum*, genetic cotton transformation, pollen-tube pathway, Cry1Ia12, *Anthonomus grandis*, *Spodoptera frugiperda*

## Abstract

*Gossypium hirsutum* (commercial cooton) is one of the most economically important fibers sources and a commodity crop highly affected by insect pests and pathogens. Several transgenic approaches have been developed to improve cotton resistance to insect pests, through the transgenic expression of different factors, including Cry toxins, proteinase inhibitors, and toxic peptides, among others. In the present study, we developed transgenic cotton plants by fertilized floral buds injection (through the pollen-tube pathway technique) using an DNA expression cassette harboring the *cry1Ia12* gene, driven by CaMV35S promoter. The T0 transgenic cotton plants were initially selected with kanamycin and posteriorly characterized by PCR and Southern blot experiments to confirm the genetic transformation. Western blot and ELISA assays indicated the transgenic cotton plants with higher Cry1Ia12 protein expression levels to be further tested in the control of two major *G. hirsutum* insect pests. Bioassays with T1 plants revealed the Cry1Ia12 protein toxicity on *Spodoptera frugiperda* larvae, as evidenced by mortality up to 40% and a significant delay in the development of the target insects compared to untransformed controls (up to 30-fold). Also, an important reduction of *Anthonomus grandis* emerging adults (up to 60%) was observed when the insect larvae were fed on T1 floral buds. All the larvae and adult insect survivors on the transgenic lines were weaker and significantly smaller compared to the non-transformed plants. Therefore, this study provides GM cotton plant with simultaneous resistance against the Lepidopteran (*S. frugiperda*), and the Coleopteran (*A. grandis*) insect orders, and all data suggested that the Cry1Ia12 toxin could effectively enhance the cotton transgenic plants resistance to both insect pests.

## Introduction

Cotton (*Gossypium hirsutum*) is an economically important crop due to lead global source of natural fiber and also contribute in oil and seed meal production. However, this worldwide crop is affected by several biotic stresses that cause a dramatic reduction in plant productivity (Oerke, 2006). Among the most important insect pest that affecting cotton crops, we can highlight *Spodoptera frugiperda* and *Anthonomus grandis* (Gallo et al., 2002; Kriticos et al., 2015). The fall armyworm, *S. frugiperda* (J. E. Smith; Lepidoptera: Noctuidae), is an important insect pest that attacks many crops. In cotton, *S. frugiperda* prefers to oviposit on the lower surface of the leaves in most plant phenological stages, which difficult the insect control by insecticides (Pitre et al., 1983; Ali et al., 1989; Fernandes et al., 2002; Miranda, 2006; Barros et al., 2010). Immediately after the eggs hatching, fall armyworm larvae start feeding on the leaf causing significant damage to the plant. On the other hand, currently, cotton boll weevil, *A. grandis* Boheman (Coleoptera: Curculionidae) is the main pest affecting cotton production in South America. During the infestation, this insect increases cotton flower bud abscission and fruit fall, especially caused by its feed establishment, mechanic damage and oviposition, which results in a significant reduction of fiber production (Santos et al., 2003). Both *S. frugiperda* and *A. grandis* can devastate entire cotton fields and the control of both can represent 25% of cotton production cost (Brazilian Ministry of Agriculture, 2015). Therefore, the need to control *S. frugiperda* and *A. grandis* infestations in cotton fields is the main cause of development and expansion of insecticide control, as well as the efforts engagement in improve genetically modified (GM) cotton varieties resistant to these insect pests.

In an attempt to control crop insect pest populations throughout the world, several GM cotton lines were developed with considerable impact to reduce losses in cotton productivity. Considering this advance, currently cotton represents the third largest GM planted area of the world, comprising 13.7% of total worldwide (James, 2014). The main features inserted into cotton plants are resistance to lepidopterans and tolerance to herbicide or a combination of both traits (James, 2014). However, none of the commercial GM cotton varieties contribute to the control of coleopteran A. grandis (ISAAA, 2015).

The majority of GM cotton plants are obtained by insertion of *cry* genes, originated from *Bacillus thuringiensis*. With almost 750 *cry* genes described and grouped into 73 classes (Crickmore et al., 2014), the crystalline inclusions produced by *B. thuringiensis* have been shown to be toxic to several insects, nematodes, mites, and protozoans (Hofte and Whiteley, 1989; Feitelson et al., 1992; Schnepf et al., 1998; Hu et al., 2010; Bravo et al., 2013; Pan et al., 2014). The Cry1 toxin is the most studied toxin class, with more than 260 genes described (Crickmore et al., 2014). Despite its specificity to lepidopterans, some of the Cry1 proteins have shown activity against coleopterans (Escudero et al., 2006; Soberón et al., 2010). Previously, Grossi-de-Sa et al. (2007) demonstrated that the recombinant Cry1Ia12 protein, identified in a *B. thuringiensis* S811 strain and expressed in *Escherichia coli* cells, was toxic to both cotton boll weevil larvae and fall armyworm (*S. frugiperda*). In addition, Guimarães et al. (2010) performed food security assays showing that Cry1Ia12 does not have any toxic effects on mice and thus could be suitable for the production of commercial GM plant varieties.

Different methods of transferring exogenous genes into cotton plants have been studied and used in recent decades. The most common techniques used for cotton transformation are *Agrobacterium*-mediated (Wu et al., 2008; Kumar et al., 2009; Mao et al., 2011; Vajhala et al., 2013) and particle bombardment (McCabe and Martinell, 1993; Rajasekaran et al., 1996; Rech et al., 2008; Rajasekaran, 2013). Other methods, including the direct delivery of DNA into protoplasts by electroporation and PEG-mediated gene transfer, have also been successfully employed (Chilton, 2005; Vain, 2007). Successful regeneration methods for cotton plants have been described (Rajasekaran et al., 1996; Leelavathi et al., 2004), although, in general, modifications are necessary when limitations to regenerate native cotton cultivars are considered (Khan et al., 2010). Plant regeneration from single transformed cells often produces somaclonal variations, which affect plant phenotypes and genotypes (Larkin, 2004). Several unwanted and unintended oscillations have been described, including point mutations, gene duplications, chromosomal rearrangements, and changes in DNA methylation (Larkin, 2004; Wilkins et al., 2004; Oh et al., 2007). These variations usually result in cotton off-types that reduce the commercial value of the generated plants. Therefore, the development of tissue-culture independent plant transformation techniques is of great interest.

To avoid these limitations, it is necessary to develop genotype-independent approaches. In this context, transformation techniques that target ovaries, meristems or other tissues, which ultimately give rise to gametes are included (Birch, 1997). The pollen-tube pathway approach represents a tissue-culture-free alternative for cotton transformation (Luo and Wu, 1989; Zhen et al., 1998; Yu et al., 1999). The genetic transformation occurs via direct delivery of foreign DNA into the pollinated and fertilized ovary (Zhou et al., 1983). This transformation method has been successfully used to introduce total exogenous genomic or plasmidial DNA into varieties of rice (Luo and Wu, 1989), soybean (Zhao et al., 1995; Liu et al., 1997), cotton (Ni et al., 1998), watermelon (Chen et al., 1998), wheat (Yu et al., 1999), onion (Peﬄey et al., 2003), and maize (Zhang et al., 2005; Yang et al., 2009).

In this present study, GM cotton plants with stable expression of Cry1I toxin were obtained, demonstrating toxicity to both cotton pests, *A. grandis* and *S. frugiperda*. The *cry1Ia12* gene was introduced into BRS Cedro cotton variety using the pollen-tube pathway technique. According to insect bioassays with floral buds of GM cotton events, the transgenic plants with a relatively high level of Cry1Ia12 toxin expression displayed insect-resistance to both insect-pests.

## Materials and Methods

### Plant Material and Culture Conditions

The cotton (*G. hirsutum* L.) elite cultivar BRS Cedro was used as recipient of a microinjection in a greenhouse at the Embrapa Genetic Resources and Biotechnology laboratory in Brasilia, Brazil. The cultivar were planted in plastic bags containing soil as substrate and maintained in a greenhouse (average temperature 26 ± 1°C; average humidity 70 ± 10%).

### Plasmid Constructs

The pCry1 vector containing the *cry1Ia12* gene under the control of 35S promoter of cauliflower mosaic virus (CaMV35S) *in tandem* with the alfalfa mosaic virus enhancer (AMV) was generated and introduced into the pCambia2300 vector. The cassette also contained the *npt*II coding sequence, which was also under the control of CaMV35S-AMV regulatory sequence. The *cry1Ia12* gene was subcloned upstream of nopaline synthase terminator (t-NOS), and the *npt*II gene, which confers kanamycin resistance, was subcloned upstream of the 35S terminator (Supplementary Figure S1A). The resistance to this antibiotic is needed to select the T0 cotton transformation events.

### DNA Application via Microinjection

The DNA application procedure described by Zhou et al. (1983) was performed with some modifications. To use the pollen-tube pathway transformation technique, pollination must be completed with consequent pollen tube development and fecundation. This process is indicated by the color of the petals, which is creamy white on the flowering day when anthesis and pollination occur; the petals turn purple on the following day (Supplementary Figure S1B1). After flowering for 24 h (the day after anthesis), young ovaries located on reproductive branches were selected, identified and tagged for microinjection. Untreated flowers were removed. The flower petals, stamen and style were carefully removed to expose the young boll and microinjection was performed (Supplementary Figure S1B2). A Hamilton microsyringe was used to inject 10 μL of plasmid DNA (0.1 μg μL^-1^) into the exposed style (Supplementary Figure S1B3). Five, ten, and twenty days after transformation, the branches were checked, and new flowers were removed. The first injections were performed 59 days after sowing. Several months later, mature bolls were harvested, and the T0 labeled seeds were removed by ginning.

### Selection and Screening of Putative Transformants

Seeds from microinjected plants were sown in plastic bags containing soil as substrate (Supplementary Figure S1B4). Ten days after seed germination, tests to kanamycin resistance were performed to select putative transgenic plants. Briefly, a cotton swab that had been wetted with a 5 μg mL^-1^ kanamycin solution was applied to the surface of the younger leaves of both transformed and non-transformed plants once a week. After 3 weeks, the leaves were examined for signs of necrosis at the sites of antibiotic application. Those leaves that did not show signs of necrosis were selected for further analysis (Supplementary Figures S1B5–B8).

### PCR Analysis of Transgenic Cotton Plants

Genomic DNA from selected cotton leaves was isolated following the procedure described by Michiels et al. (2003) with some modifications. The presence of *cry1Ia12* was confirmed using the primers *cry1Ia12* forward (5′-ACGCCAAGGTTGACAAAATC-3′) and *cry1Ia12* reverse (5′-AGGGAGCTTCTGAACGAACA-3′) to amplify a 420 bp internal fragment, denominated by *cry1Ia12* segment (ICS). The reaction was performed with 100 ng of DNA as follows: an initial denaturation at 95°C for 5 min followed by 32 cycles of denaturation at 95°C for 1 min; annealing at 55°C for 30 s; and extension at 72°C for 1 min, followed by a final extension for 10 min at 72°C. DNA from a non-transgenic *G. hirsutum* plant was used as the negative control, and the pCry1 vector used as positive control.

### Evaluation of the Integrated DNA Using Southern Blot Analysis

Total genomic DNA from the leaves of non-transgenic and transformed cotton plants was isolated using a CTAB method modified from Michiels et al. (2003). Fresh leaves (1 g) were ground to a powder in liquid nitrogen, which was directly transferred into 15 mL of extraction buffer preheated to 60°C. The suspension was mixed carefully and incubated at 60°C for 60 min. After an extraction step using chloroform-iso-amyl-alcohol (24:1), an overnight isopropanol precipitation step at 25°C was performed. The following washing steps were performed as described by Michiels et al. (2003). Once ethanol-free, the DNA pellet was dissolved in sterile water, which was incubated with RNase (100 μg mL^-1^) at 37°C for 2 h. The DNA purification was performed according to the manufacturer’s instructions from DNAeasy extraction Kit (QIAgen^®^). The genomic DNA quantification and purity ration were determined using a NanoVue spectrophotometer (GE Healthcare Life Science^®^).

Twenty micrograms of genomic DNA was digested with *Nco*I and *Hind*III restriction enzymes. The digested DNA was resolved in 0.8% agarose gel electrophoresis and then transferred onto a nitrocellulose membrane (GE Healthcare Life Science^®^). A 2234 bp *cry1Ia12* DNA fragment was the probe used in hybridization step, which was labeled with α-[32P]-dCTP using a Random Primer DNA Labeling kit (Ready-to-Go DNA labeling beads, GE Healthcare Life Science^®^). Hybridization was performed at 65°C for 16 h, and the filter was washed at room temperature with 2x SSC/0.1% SDS and 1x SSC/0.1% SDS for 15 min each and at 60°C with 0.2x SSC/0.1% SDS for 15 min (Sambrook and Russell, 2001). After washing steps, the membrane was exposed to an imaging plate (BAS-MP, FujiFilm^®^) for 24 h. Images were acquired using a FLA3000 phosphoimage (FujiFilm^®^).

### Qualitative Cry1Ia12 Protein Analysis in Cotton Leaves

The Cry1Ia12 protein expression was analyzed in cotton plants using Western blot assays. Approximately 3 g of leaves from transgenic and non-transgenic plants were pulverized in a mortar in liquid nitrogen with a pestle until a fine powder was obtained. Proteins from the leaves were then homogenized in pre-chilled protein extraction buffer (50 mM Tris at pH 7.4, 150 mM NaCl, 10 mM sodium metabisulfite, 0.2% ascorbic acid, 0.1 M EDTA and 0.5% Triton) at 4°C. The extracts were centrifuged at 10,000 × *g* for 10 min at 4°C, and the supernatant was quantified using the Bradford protein assay (Bio-Rad^®^). A polyacrylamide gel (7.5%) was loaded with 100 μg of protein samples and approximately 500 ng of purified Cry1Ia12 at 20 mA. The protein gel was electroblotted at 10 V for 30 min onto a nitrocellulose membrane (Hybond-C^®^ Extra, Amershan Biosciences^®^) using a Trans-Blot SD Semi-Dry cell (Bio-Rad^®^). After transfer, the nitrocellulose membrane was blocked using a TBS buffer containing 1% gelatin and 0.25% PVA (polyvinyl alcohol) and then probed with an anti-Cry1Ia12 polyclonal antibody produced in rabbits (Genescript^®^). Goat anti-rabbit antibodies conjugated to alkaline phosphatase (SIGMA^®^) were used to detect the Cry1Ia12 protein. The reactive protein in the nitrocellulose membrane was revealed using an AP conjugate substrate kit (Bio-Rad^®^) according to the manufacturer’s instructions.

### Quantification of Expressed Cry1Ia12

To quantify the Cry1Ia12 in cotton leaves, an indirect ELISA (Engvall and Perlmann, 1971) was performed with 2 μg of the total protein extracted from each selected transgenic and a non-transgenic cotton plants. The preference for leaves was based on specific reasons: (i) in the case of *A. grandis*, the insect has its preferred feeding site in the vicinity of this tissue, precisely because this insect species is highly selective; (ii) in the case of *S. frugiperda*, the main objective was controlling this insect populations in early larval instars, where feeding occurs preferably on leaves.

The assay was performed in triplicate on a high-binding 96-well EIA/RIA microplate (Costar^®^ 3590). A standard curve was obtained using purified Cry1Ia12. The plate was incubated with anti-Cry1Ia12 polyclonal antibody (Genescript^®^) and then incubated with goat anti-rabbit antibody conjugated to HRP. The plates were washed and the substrate solution was added to each well. The reaction was stopped after 30 min by the addition of 50 μL of 2 M sulfuric acid. The assay was read on a SpectraMax 190 microplate reader (Molecular Devices) at 450 nm.

### Insect Bioassays

Transgenic cotton plants from the T1 progeny were subjected to bioassays with cotton boll weevils and fall armyworms. Eggs of *A. grandis* and *S. frugiperda* species were provided by the Bioecology and Semiochemicals of Insects Laboratory at Embrapa Genetic Resources and Biotechnology at Brasilia, DF, Brazil. Both *A. grandis* and *S. frugiperda* adults were maintained in an environmental controlled room with 26 ± 2°C with controlled humidity of 70 ± 10%. The insects were maintained with artificial diet, according to its specificity. The eggs were collected and then separated into petri dishes with the same diet as the adults were fed (Greene et al., 1976; Martins et al., 2007). All the experiments were performed with biological (each distinct bioassay performed at different periods of time) and technical (all experimental repetition performed during each bioassay) triplicates. The data were statistically analyzed using *ANOVA*.

#### *Spodoptera frugiperda* Bioassay

Concerning *S. frugiperda* bioassay, the eggs were placed in non-transgenic cotton leaves. First instar larvae that hatched remained feeding on these non-transgenic leaves for 2 days when they reached the second instar stage. Ten fully expanded cotton leaves were detached from each non-transgenic and transgenic cotton plant of the T1 generation and placed in an entomology test chamber. One second instar larva was released onto each plate and allowed to feed on the leaf. The plates were kept at 25–27°C with controlled humidity of 70 ± 10%. Data on the survival and weight of each living larva were recorded on the 10th day.

#### *Anthonomus grandis* Bioassay

Plants containing 6 mm flower buds were selected for the boll weevil bioassays. A population of *A. grandis* was maintained at the Insect Rearing Platform at Embrapa Genetic Resources and Biotechnology on a standard rearing diet at 27 ± 2°C, 70 ± 10% relative humidity, and a photoperiod of 14 h (Monnerat, 2000). One *A. grandis* egg containing an active embryo was inoculated in a 6 mm cotton flower bud. Bud perforation was performed using a drill, and the orifice was sealed with vaseline to prevent egg dehydration. The experimental period was 20 days. After this, the mortality rate and the adult’s weight were measured.

## Results

### Pollen-Tube Pathway Transformation and Selection of Transgenic Cotton Plants

A total of 590 floral buds were microinjected, among which 315 were aborted due to the mechanical process of microinjection. The 275 remaining floral buds produced 3175 cotton seeds, which were planted in soil bags and maintained in a greenhouse. After antibiotic selection a total of 43 plants showed no signs of necrosis, indicating the presence of *npt*II gene. These plants were used in further analyses.

### Molecular Characterization and Quantitation of Cry Toxin in Transgenic Cotton Plants

**FIGURE 1:**
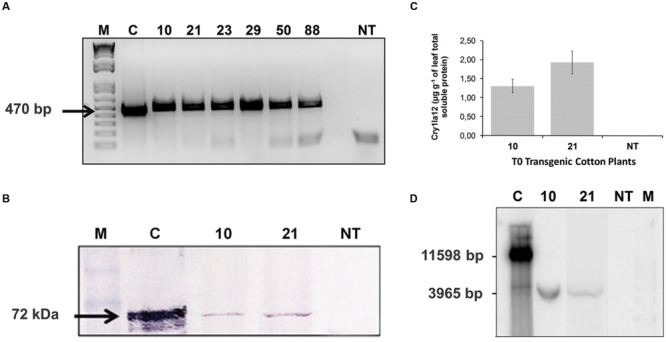
**Molecular analysis of T0 transgenic cotton plants. (A)** Amplicon with 470 bp obtained by PCR amplification of *cry1Ia12* gene fragment presented in T0 cotton plants, which were selected in medium with kanamycin. **(B)** Detection of Cry1Ia12 protein in Western blot assay. **(C)** Indirect ELISA to Cry1Ia12 protein quantification in two T0 cotton plants; **(D)** Southern blot showing integration of DNA cassette with *cry1Ia12* gene into cotton genome. *Legend:* The numbers 10, 21, 23, 29, 50, and 88 in the four panels are the identifiers of T0 GM cotton plants, as well as *NT* is the non-transformed plant. In **(A,D)** *M* represents the DNA molecular marker (1 kb Plus DNA Ladder – Invitrogen^®^ – CAT. 10787-018) and *C* is the positive control (pCry1 vector). In **(B)**, *M* is the protein molecular marker (BenchMark^TM^ Pre-stained Protein Ladder – Invitrogen^®^ – CAT. 10748-010) and *C* represents a positive control (heterologous Cry1Ia12, expressed in *Escherichia coli*; Grossi-de-Sa et al., 2007).

The *cry1Ia12* segment (ICS) was amplified by PCR technique, and according to **Figure 1A**, the T0 plants numbered 10, 21, 23, 29, 50, and 88 (lanes 2–3) were positive for this amplification. Regarding Cry1Ia12 protein expression in T0 cotton plants, immunoblots using an anti-Cry1Ia12 polyclonal antibody revealed that the Cry1Ia12 protein with approximately 72 kDa was expressed at significant levels only in two GM cotton plants, 10 and 21, which were 1.25 and 2.26 μg g^-1^ of leaf respectivelly (**Figures 1B,C**). For this reason, the following molecular experiments were performed with these two GM events. Therefore, Southern blot analysis were carried out in order to confirm the DNA cassette integration in the cotton genome of these two events (10 and 21; **Figure 1D**). The blot suggests a successful cassette genome integration in both events, which is in accordance with other molecular assays, especially with PCR experiments.

**FIGURE 2:**
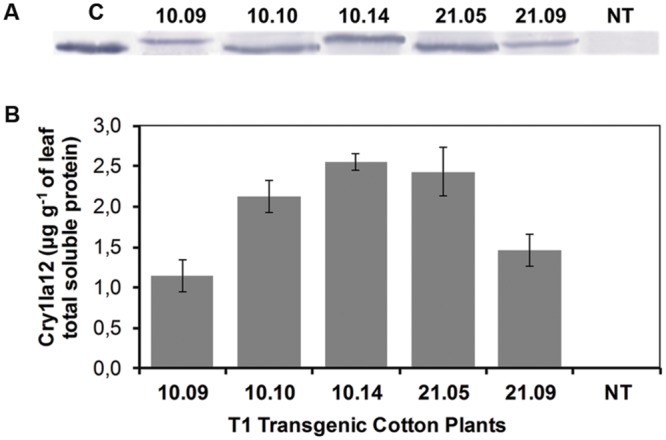
**Molecular analysis of T1 transgenic cotton plants. (A)** Western blot assay to detect Cry1Ia12 protein in 10 and 21 progeny; **(B)** Quantification of Cry1Ia12 protein by indirect ELISA. *Legend:* The numbers 10.09, 10.10, 10.14, 21.05, and 21.09 in the two panels are the identifiers of T1 GM cotton plants (10 and 21 progeny), as well as *NT* is the non-transformed plant and *C* represents a positive control (heterologous Cry1Ia12, expressed in *Escherichia coli*; Grossi-de-Sa et al., 2007).

The T1 progeny of both 10 and 21 T0 GM cotton plants were molecularly evaluated and five of them (10.09, 10.10, 10.14, 21.05, and 21.09) were chosen based on both Western blot and ELISA experiments to biossays with *S. frugiperda* and *A. grandis*. The 10.14 T1 cotton plant demonstrated higher protein level (∼2.56 μg g^-1^ of leaf) when compared to respective T0 parental event (**Figure 2**).

### Insect Bioassays of Transgenic Plants

#### The Transgenic Cotton Plants Exhibited Toxicity to *Spodoptera frugiperda*

**FIGURE 3:**
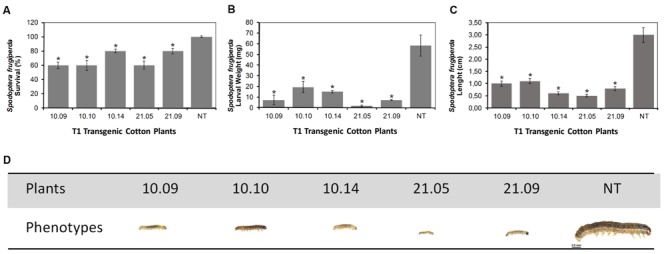
**Bioassays of transgenic cotton plants with *Spodoptera frugiperda*.** The analysis was performed on the T1 transgenic plants with the non-transgenic plants acting as controls. **(A)** The survival of *S. frugiperda* larvae. **(B,C)** Plots with weight and length of the larvae after feeding, respectively; **(D)** A schematic diagram representing the larvae lenght (phenotypes) of different cotton transformed T1 plants. The survival rate was determined on the 10th day after inoculation. *Legend:* The numbers 10.09, 10.10, 10.14, 21.05, and 21.09 in the four panels are the identifiers of T1 GM cotton plants (10 and 21 progeny), as well as *NT* is the non-transformed plant. The asterisks (^∗^) highlight the samples with significant statistical difference (ANOVA, *p* ≤ 0.05) using the NT cotton plant as the reference.

Five transgenic T1 plants (10.09, 10.10, 10.14, 21.05, and 21.09) were evaluated for their toxicity to cotton fall armyworm larvae. Initially the insects were fed with non-transgenic leaves for 2 days, and then they were transferred to transgenic leaves for 10 days. During the first 5 days, the experiment showed that the transgenic cotton plants were more resistant to *S. frugiperda*, compared to untransformed control, due to the fact that leaves have been slightly ingested by the larvae (**Figure 3**). Amongst the 6th and 10th days, nearly half of the leaves in the control plant had been consumed, while the transgenic leaves were barely fed. The lenght from control larvae were significant larger than the ones that had fed on transgenic plants (Supplementary Figure S2). Besides the minor damage to the leaves, a significant delay in the larvae growth fed on GM transformed plants were observed. Although, in terms of mortality, all 21 GM cotton plant progeny have shown greater toxicity to *S. frugiperda*, the five GM cotton plant progeny had significantly influenced the larvae development. All surviving larvae presented smaller lenght and were extremely weak, compared to larvae fed on non-transformed plants. The evaluated data showed that Cry1Ia12 toxin expressed in GM cotton plants was toxic to the cotton fall armyworms, as evidenced by larvae mortality rate up to 40%, after 10 days of experimental evaluation (**Figure 3A**). In the control insect group, all the larvae survived weighed approximately 60 mg (**Figure 3B**), while the weights of the surviving larvae fed on transgenic cotton plants ranged from 2 to 15 mg after 10 days of feeding, demonstrating be extremely smaller and weaker (**Figures 3C,D**), and obviously committing the next generation of the insect population.

#### The Transgenic Cotton Plants Exhibited Toxicity to *Anthonomus grandis*

**FIGURE 4:**
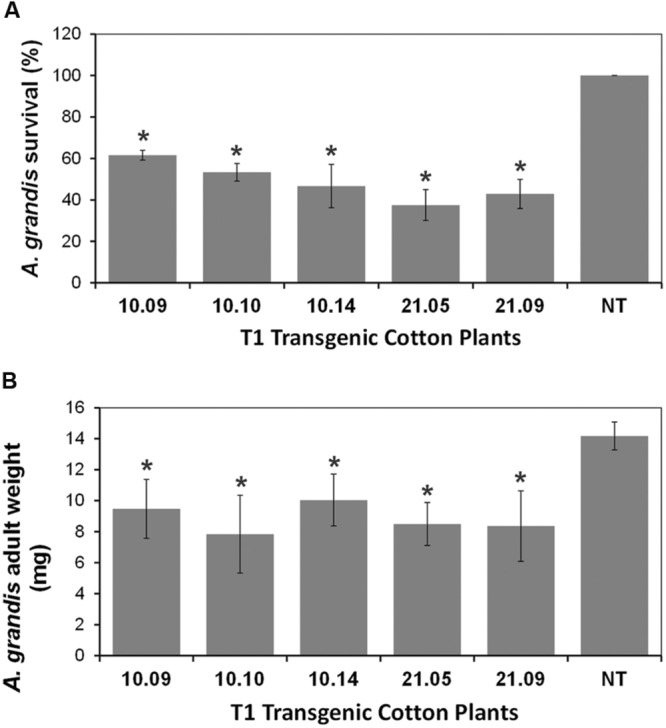
**Bioassays of Transgenic Cotton Plants with *Anthonomus grandis*.** This analysis was performed on the T1 transgenic plants with the non-transgenic plants acting as controls. **(A)** The survival of *A. grandis.* **(B)** The adults weight. Bioassays were conducted inoculating *A. grandis* eggs into a 6 mm cotton flower bud and survival rate was determined on the 20th day after inoculation. *Legend:* The numbers 10.09, 10.10, 10.14, 21.05, and 21.09 in the four panels are the identifiers of T1 GM cotton plants (10 and 21 progeny), as well as *NT* is the non-transformed plant. The asterisks (^∗^) highlight the samples with significant statistical difference (ANOVA, *p* ≤ 0.05) using the NT cotton plant as the reference.

The same T1 cotton plants expressing Cry1Ia12 used in *S. frugiperda* bioassay were also evaluate to their ability to confer resistance against to the cotton boll weevil. A total of ten floral buds were subjected to *A. grandis* bioassays. Once all the eggs have hatched is expected that the Cry1Ia12 protein expressed in the GM cotton plants do not block the hatching process. This statement is based on the fact that the toxin needs to be processed in the *A. grandis* midgut to become active (Schnepf et al., 1998), which emphasizes the necessity that Cry proteins must be ingested by the insect to have activity. On the other hand, after 7 days, it was feasible to evaluate whether the larvae had fed, according the floral buds phenotypes, which became “fluffy,” in case of feeding. This feeding pattern was evident in all non-transgenic plants. Compared with the untransformed, the transgenic plants expressing the Cry1Ia12 toxin showed substantially less damage to the floral bud after a week of feeding. The boll weevils completed their development and became adults in all non-transgenic plants after 20 days. Amongst transgenic plants, the number of adults that emerged was less than those emerged from control. Development delay in some groups was also observed (Supplementary Figure S3). The mortality of boll weevils in the experimental groups reached up to 60% after 20 days (**Figure 4**), which was significantly higher than control group. All the larvae survived became adult insect on non-transgenic plants. The emerged insects that feed on floral buds from cotton line 10 progeny had a lower mortality rate than those which feed on the cotton line 21 progeny (**Figure 4**).

## Discussion

The plant transformation primary goal is the production of fertile plants expressing a desirable foreign gene. To generate plants that express the desired traits, different techniques for inserting genes into plants have been developed since the early 1980s (De Block et al., 1984) and are currently widely used and commercially available (James, 2014). Cotton transformation methods normally use co-cultures with *A. tumefaciens* (Guo et al., 2007) and microprojectile bombardment (Aragão et al., 2005). It is well known that the transformation efficacy is affected by the plant material, genotype/variety, and type of explant (Wilkins et al., 2004; Rao et al., 2009; Anami et al., 2013; Chakravarthy et al., 2014; Juturu et al., 2015).

The pollen-tube pathway technique has been successfully used to transfer exogenous DNA into several plant species (Luo and Wu, 1989; Ni et al., 1998; Zhen et al., 1998; Yu et al., 1999). Herein, the establishment of a reliable and repeatable protocol for the pollen-tube pathway transformation technique was demonstrated, contributing to the generation of a GM Brazilian cotton variety, which expresses the Cry1Ia12 toxin that confers toxicity to the two important economic cotton insect pests, *A. grandis* and *S. frugiperda*. The pollen-tube pathway technique does not require tissue culture, which is the greatest advantage. In this context, the efficiency improvement observed with this technique relies on the seeds obtained from microinjected floral buds. In China, this transformation technique is usually applied on large scales to produce a vast number of seeds in the field (Huang and Wang, 2002). In contrast, Brazilian legislation is extremely restricted regarding to field experiments using GM plants. Therefore, such trials must be performed under greenhouse conditions, which decrease both the number of plants that can be microinjected and the seed yield. The pollen-tube pathway was efficient in transforming BRS Cedro cotton variety, reaching an efficiency of 0.01%, evaluated by the number of positive GM plants and viable seeds, as determined by PCR, Southern blot, ELISA and and Western blot assays. This is important and justify the low number of cotton plants positively featured in this work, even starting from a large initial number of ovules.

Variations in gene expression in different transformation events have been reported in other studies (Deroles and Gardner, 1988; Robert et al., 1989) and could be due to variations in the transgene’s integration into the target genome (Deroles and Gardner, 1988; Meyer, 1995a,b). Gene delivery strategies have also been explored to optimize the pollen-tube pathway technique. In cotton, the large size of the flowers allows for injection into an ovary.

According to Monsanto (2002), Cry1Ac protein content of Bollgard I cotton leaves was around 1.56 μg g^-1^ of leaf total soluble protein. Comparing the Bollgard I Cry1Ac expression levels with the cotton 10 and 21 progenies that best express Cry1Ia12 protein (10.14 and 21.05, respectively), it can be seen nearly the same toxin expression levels in both Cry1Ia12 cotton plants. Thus, this observation can be explained by several ways, highlighting: (i) intrinsic characteristics of each *cry* gene; (ii) the transgene insertion site in cotton genome, and (iii) gene promoter activity. Thus, in future studies is intended to use gene promoters that provide higher levels of expression, as well as presented by *uce* promoter, identified by Viana et al. (2011), which drive high expression in root and flower cotton tissues. Besides, several new gene promoters induced by biotic stress can be identified and characterized, especially after data analysis obtained from transcriptome of cotton flower buds infested with *A. grandis* larvae (Artico et al., 2014).

Since cotton was first transformed by two distinct groups in 1987 (Firoozabady et al., 1987; Umbeck et al., 1987), different traits have been introduced into cotton plants aiming either abiotic tolerance or biotic resistance (Juturu et al., 2015). The use of plant transformation to control insect pests started, when Perlak et al. (1990) developed transgenic cotton expressing the *B. thuringiensis* toxin Cry1Ac. After this achievement, GM cotton harboring *cry* genes to control insect pests have been available (James, 2014).

Once it was determined that Cry toxins are responsible for insect resistance in most GM plants, several studies were performed to evaluate the role of these toxins in response to insect stress. Tabashnik et al. (2002) showed that the Cry1Ac-resistant pink bollworm (*P. gossypiella*) had little or no ability to survive on second-generation transgenic cotton containing Cry2Ab alone or Cry1Ac plus Cry2Ab. Bioassays of several independent transgenic maize lines over-expressing the *cry1Ie* gene showed that these transgenic plants were highly toxic to the wild-type cotton bollworm (*Helicoverpa armigera*), producing mortality levels of 50% after 6 days of exposure (Zhang et al., 2013).

Even though the main class of genes used to obtain GM cotton resistant to insects, members of the *cry* family have limitations, mainly associated with molecular activities mechanisms. According to literature data, the active toxins bind with specific receptors on the brush border membrane of gut epithelial cells and is partially inserted into the membrane, generating pores. This results in colloid osmotic lysis of gut epithelial cells followed by the death of the insect (Hofmann and Lüthy, 1986; Schnepf et al., 1998). The most common resistance mechanism is a reduction of the toxin’s ability to bind to its specific midgut receptor(s). This may also confer cross-resistance to other toxins that share the same receptor (Ferré and Van Rie, 2002). In order to overcome this problem, various *cry* genes homologs have been characterized for insecticide function. According to specific studies, the Cry1I toxins group (where Cry1Ia12 is inserted) has wide host range and was initially characterized by their dual activity toward Lepidoptera and Coleoptera (Escudero et al., 2006). Among them, Martins et al. (2008) demonstrated in bioassays with heterologous Cry1Ia protein (expressed in baculovirus) in artificial diet that the recombinant protein had toxicity to *S. frugiperda* and *A. grandis* larvae. In parallel, Grossi-de-Sa et al. (2007) highlighted the *cry1Ia12* importance to *A. grandis* and *S. frugiperda* populations control in artificial diet. This feature has made it possible to test the susceptibility of *A. grandis* and *S. frugiperda* to a heterologous Cry1Ia-type toxin (Cry1Ia12 expressed in *Escherichia coli*) and demonstrated that the Cry1Ia12 toxin kills both insect larvae in concentration of 230 and 5 μg mL^-1^ of artificial diet, respectively. The current study and those presented by Grossi-de-Sa are complementary. In both studies, *A. grandis* and *S. frugiperda* populations were controlled and they were differentiated by toxin administration (artificial diet and GM cotton plant). Thereby, assuming that Cry1Ia type protein sequences do not differ much from one another (Cry1Ia12 shows 99% of identity and similarity with other Cry1Ia toxins deposited in databases; Grossi-de-Sa et al., 2007), it is possible to suggest that the present study corroborates with others concerning the control of the cotton boll weevil and fall armyworm populations with Cry1Ia toxin variants administration.

Several studies showed a gradually increase in the number of insect populations resistant to Cry toxins. Downes et al. (2010) demonstrated that *Helicoverpa* species (Noctuidae) were resistant to Cry2Ab toxin in the second generation of *B. thuringiensis* cotton. Additionally, the emergence of *S. frugiperda* populations resistant to the *B. thuringiensis* toxin Cry1Fa expressed in corn was noted, forcing producers to use pesticides to reduce the damage caused by this insect pest (Monnerat et al., 2015). In order to retard the process of resistance, researchers postulate that transgenic plants should express high doses of the toxin or use more than one gene of interest in genetic transformation. Theoretically, a plant with two transgenes is significantly more effective in controlling insect pests than those expressing only one, since the insect that is resistant to a first toxin could likely be killed by a second (Roush, 1998; Zhao et al., 2005). Liu et al. (2005) demonstrated that *H. armigera* larvae in the first, second, and third instar could not survive if fed on transgenic cotton leaves expressing Cry1A + CpTI (Cowpea Trypsin Inhibitor) and Cry1Ac. In this present study, bioassays using *S. frugiperda* and *A. grandis* showed that cotton plants expressing Cry1Ia12 were toxic to both of these insect pests, but its insecticidal activity could be enhanced by associating with other molecules (Cry or non-Cry). Among them, we can point out: Cry8-type toxins (Nakasu et al., 2010; Oliveira et al., 2011; Navas et al., 2014), trypsin/chymotrypsin inhibitors (Franco et al., 2003; de Pg Gomes et al., 2005; Cruz et al., 2013), alpha-amylase inhibitors (Oliveira-Neto et al., 2003; Dias et al., 2005; Bonavides et al., 2007) and *Streptomyces* cholesterol oxidase (Purcell et al., 1993). Moreover, it is worth emphasizing that gene knockdown by dsRNA technology has been widely used to silence important insect genes. A great example would be the *A. grandis* chitin synthase I (*AgCHI*) knockdown, that resulted in normal oviposition of unviable eggs and malformed alive larvae that were unable to develop in artificial diet (Firmino et al., 2013). Therefore, it would be also possible to associate the Cry1Ia12 toxin with dsRNA molecules in order to increase the control of *A. grandis* and *S. frugiperda* populations. Thus, Cry1Ia12 GM cotton plants can be used in breeding strategies to obtain GM cotton lines more effective in pest control, as well as presenting a reduction in emergence of resistant insects, especially *S. frugiperda*.

Considering, until now, the absence of cotton varieties with natural resistance to *A. grandis* infestation, as well as the great financial losses caused by this insect to cotton culture, we emphasize that the Cry1Ia12 GM cotton plants presented in this work are the first step in effective pesticide-free combat to this insect pest, even if the *A. grandis* mortality rate is still far from adequate. This observation is based mainly on the high cost of insecticides, besides the negative environmental impacts caused by these chemical agents. In Brazil, for example, the cost of insecticides to combat the boll weevil infestation in cotton crops ranged in 2015 between US$ 100 and US$ 300 per hectare (45% increase compared to last year; Brazilian Ministry of Agriculture, 2015). Thereby, a 60% mortality rate would be already significant, because would reach a large reduction in the cost of cotton production and consequently in insecticide management. Therefore, these GM cotton plants also present high potential *A. grandis* control and can be used in breeding programs to reduce the damage caused by this insect pest to cotton culture.

## Author Contributions

RdO carried out all experiments and data analysis. LdM and WL contributed to the bioassays design. FA contributed in achieving the ELISA experiments and help draft manuscript. OO-N helped establish the cotton transformation conditions. HM established the total protein extraction and Western-blot conditions. MdS, IL-T, and AB set the experimental conditions of PCR and Southern blot. MG conceived the study, planned the experiments, and helped draft the manuscript. All authors read and approved the final manuscript.

## Conflict of Interest Statement

The authors declare that the research was conducted in the absence of any commercial or financial relationships that could be construed as a potential conflict of interest.

The reviewer OF declares that, despite being affiliated with the same institute as the authors RO and MG, the review process was handled objectively.
