# Supplementary material for: Transgenic Cotton Plants Expressing Cry1Ia12 Toxin Confer Resistance to Fall Armyworm (Spodoptera frugiperda) and Cotton Boll Weevil (Anthonomus grandis)
Source: Front Plant Sci. 2016 Feb 19;7:165. doi: 10.3389/fpls.2016.00165 (PMC4759279; doi:10.3389/fpls.2016.00165)
Supplement: Supplementary file 1 [file Data_Sheet_1.PDF]

Supplementary Material

Transgenic cotton plants expressing Cry1Ia12 toxin confer resistance to fall armyworm (*Spodoptera frugiperda*) and cotton boll weevil (*Anthonomus grandis*)

Raquel Sampaio de Oliveira1,2, Osmundo Brilhante Oliveira-Neto2,3, Hudson Fernando Nunes Moura2,4, Leonardo Lima Pepino de Macedo2, Fabrício Barbosa Monteiro Arraes2,5, Wagner Alexandre Lucena2,6, Isabela Tristan Lourenço2, Aulus Anjos de Deus Barbosa2, Maria Cristina Mattar da Silva2, Maria Fatima Grossi-de-Sa1,2*

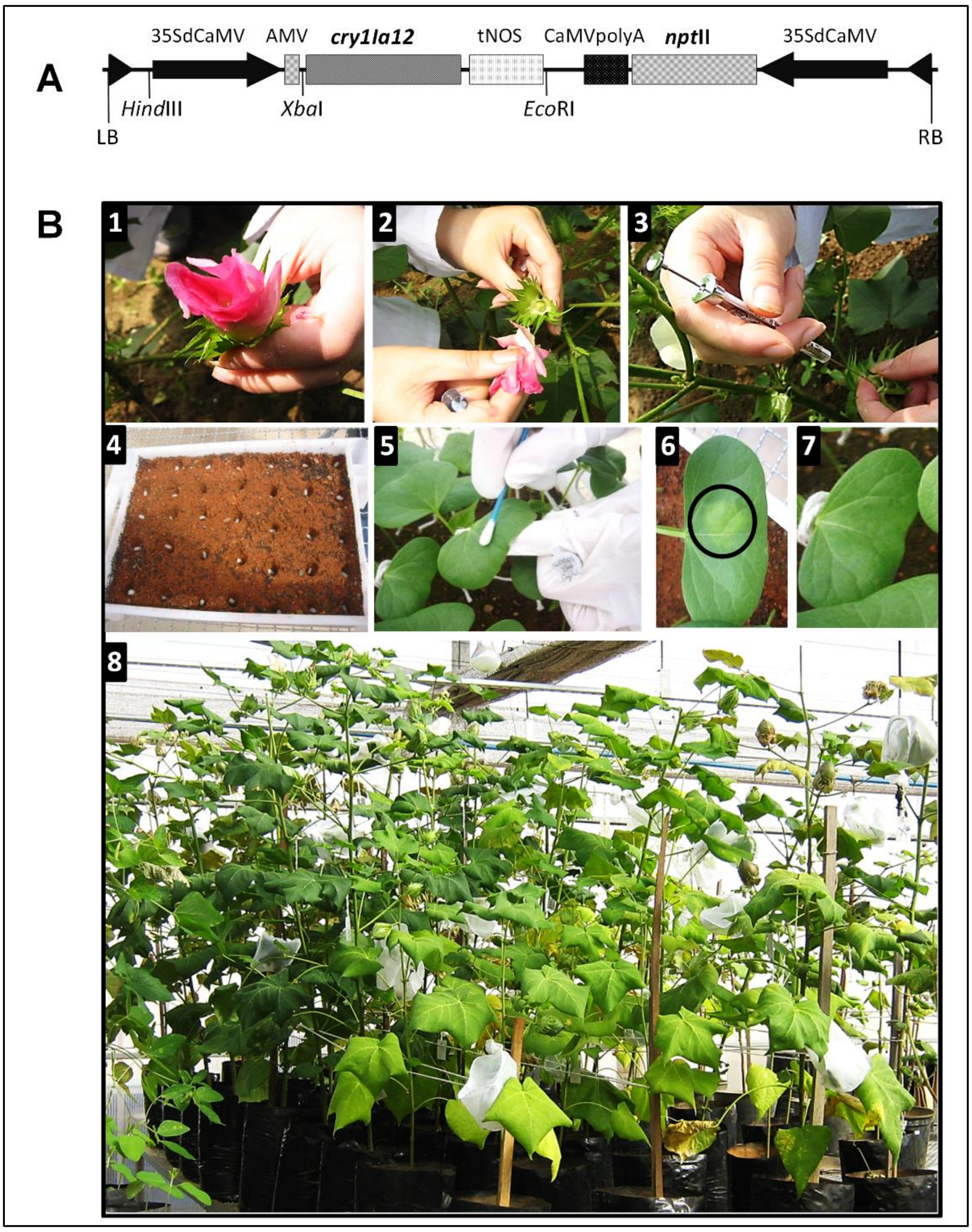

Supplementary Figure 1. Cry1Ia12 Cotton Transformation. (A) A schematic diagram of pCambia Binary Vector T-DNA. LB, Left Border; 35SdCaMV Cauliflower Mosaic Virus 35S duplicated promoter; *npt*II, Neomycin Phosphotransferase II; Tnos – NOS Terminator; RB, Right Border; (B) Steps in the pollen-tube pathway transformation for cotton plants; (B1) Identification of a pollinated cotton flower. Pollinated flowers have purple petals; (B2) Carpel exposal by removing the petals, stamen and style; (B3) Pollen tube transformation; (B4-7) Putative transformed plants were selected, seeds were sown on a tray (B7) and, after ten days, the germinated plantlets were subjected to a kanamycin assay (B5). The negative plants are identified with a chlorotic mark (B6), whereas the transformed plants have no kanamycin mark (B7); (B8) the selected transformed plants.

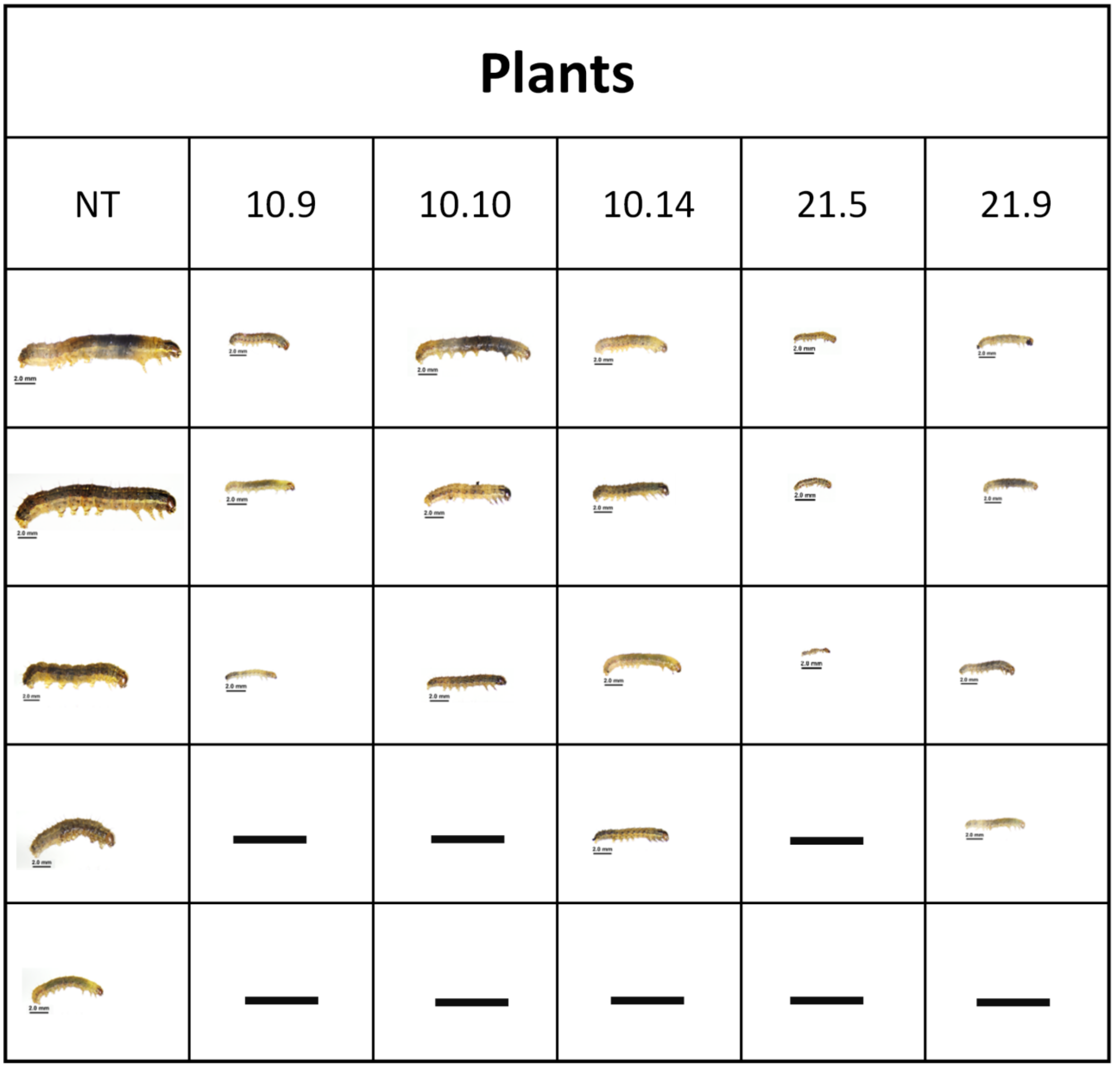

Supplementary Figure 2. **<u>Bioassay with *Spodoptera frugiperda*.</u>** Each fresh leaf from the transgenic and non-transgenic plants was infested with one 2nd instar larva. Bioassays were performed in entomological chambers. The pictures represent the surviving cotton fall armyworms that had been fed leaf samples for 10 days. Black lines represent fall armyworms that died after feeding.

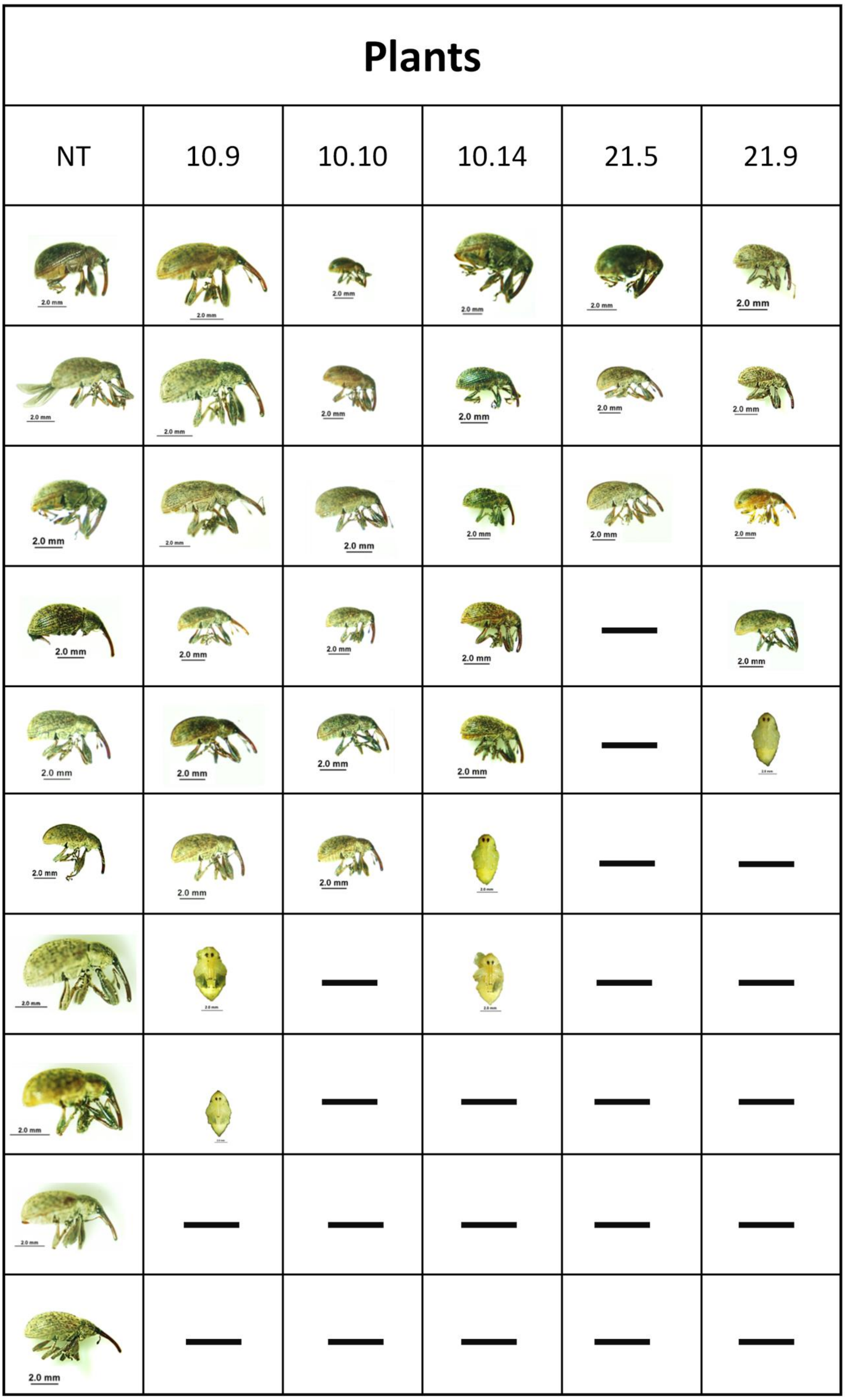

Supplementary Material Figure 3. <u>Bioassay with *Anthonomus grandis*.</u> Boll weevil eggs were placed in cotton flower buds. After 19 days, all the adults emerged from the non-transgenic cotton (NT) plants. Black lines represent *A. grandis* individuals that died after feeding.
